# High Salt Diet Affects Renal Sodium Excretion and *ERRα* Expression

**DOI:** 10.3390/ijms17040480

**Published:** 2016-04-01

**Authors:** Dan Wang, Yang Wang, Fu-Qiang Liu, Zu-Yi Yuan, Jian-Jun Mu

**Affiliations:** Department of Cardiovascular Medicine, First Affiliated Hospital of Medical College of Xi’an Jiaotong University, Key Laboratory of Molecular Cardiology, No. 277 Yanta West Road, Xi’an 710061, China; m15991631129@163.com (D.W.); wangyangxxk@126.com (Y.W.); Liufuqiang0909@163.com (F.-Q.L.); zuyiyuan@mail.xjtu.edu.cn (Z.-Y.Y.)

**Keywords:** sodium, potassium, estrogen-related receptor α, salt sensitive

## Abstract

Kidneys regulate the balance of water and sodium and therefore are related to blood pressure. It is unclear whether estrogen-related receptor α (ERRα), an orphan nuclear receptor and transcription factor highly expressed in kidneys, affects the reabsorption of water and sodium. The aim of this study was to determine whether changes in the expressions of ERRα, Na^+^/K^+^-ATPase and epithelial sodium channel (ENaC) proteins affected the reabsorption of water and sodium in kidneys of Dahl salt-sensitive (DS) rats. SS.13BN rats, 98% homologous to the DS rats, were used as a normotensive control group. The 24 h urinary sodium excretion of the DS and SS.13BN rats increased after the 6-week high salt diet intervention, while sodium excretion was increased in DS rats with daidzein (agonist of ERRα) treatment. ERRα expression was decreased, while β- and γ-ENaC mRNA expressions were increased upon high sodium diet treatment in the DS rats. In the chromatin immunoprecipitation (CHIP) assay, positive PCR signals were obtained in samples treated with anti-ERRα antibody. The transcriptional activity of ERRα was decreased upon high salt diet intervention. ERRα reduced the expressions of β- and γ-ENaC by binding to the *ENaC* promoter, thereby increased Na+ reabsorption. Therefore, ERRα might be one of the factors causing salt-sensitive hypertension.

## 1. Introduction

Salt intake is an important environmental risk factor of hypertension. Different individuals respond differently in blood pressure to salt loading or salt restriction, which is known as salt sensitivity. It is an intermediate genetic phenotype of hypertension [1]. Renal transplantations in animals and humans have revealed that kidney is the key determinant of blood pressure in response to salt intake [2,3]. In recent years, many studies have confirmed that aberrant gene expressions are associated with sodium excretion involved in the development of salt-sensitive hypertension [4,5,6]. In addition, the relationships among sodium ion transport, metabolism and salt sensitivity have also been reported [7]. However, the molecular mechanism of salt-sensitivity has not been elucidated.

Estrogen-related receptors (ERRs) are the first group of known orphan nuclear receptors [8] and are generally recognized as transcription factors [9,10]. Researchers have used chip-on-chip and DNA microarrays to search for functional ERRs, and identified their target genes. Meanwhile, gene expression analysis of ERRα knockout rats has revealed many genes associated with ERRα downstream metabolism [11,12,13]. Recent studies have discovered that ERRα is closely related to cardiovascular diseases. Sumi *et al.* [14] have shown that ERRα can be directly combined with the endothelial nitric oxide synthase (eNOS) promoter sequence to upregulate the expression of mRNA and protein levels of eNOS and stimulate the synthesis of NO. Tremblay *et al.* [15] have demonstrated that ERRα regulates the expression of channels involved in renal Na^+^ and K^+^ handling. These experiments suggest that ERRα is related to renal sodium potassium balance and blood pressure.

Although ERRs regulate nuclear-encoded genes involved in all aspects of mitochondrial functions [16], little is known about the mechanisms and signals governing the blood pressure. We hypothesize that the abnormal expression of ERRα in kidneys is key to the metabolic disorder of sodium in salt sensitive hypertension. In this study, Dahl salt sensitive rats (DS) were given diet intervention and examined for the level of renal ERRα, Na^+^/K^+^-ATPase, and epithelial sodium channel (ENaC) mRNAs and proteins. Chromatin immunoprecipitation (CHIP) assay was also used to determine the roles of ERRα, Na^+^/K^+^-ATPase and ENaC, and whether changes in the expressions of these proteins contribute to the changes in the reabsorption of water and sodium to affect the blood pressure.

## 2. Results

### 2.1. Effects of Salt Loading on Systolic Blood Pressure and Weight

All rats completed the intervention trial in normal physiological activities. There were no differences in blood pressure and weight among groups at baseline. As shown in Table 1, after high salt intervention, the average weight of both the SS.13BN rats and the DS rats was lower than that of the rats under normal salt diet interventions (309.13 ± 6.05 *vs.* 340.60 ± 2.82 g, *p* < 0.001; 313.4 ± 5.63 *vs.* 344.83 ± 8.64 g, *p* < 0.001). The blood pressure of rats in the SH (SS + High salt diet) group was higher than that of the SN (SS + Normal diet) group (174.71 ± 2.21 *vs.* 146.14 ± 2.88 mm Hg, *p* < 0.001) and that of the BH (SS.13BN + High salt diet) group (174.71 ± 2.21 *vs.* 146.43 ± 3.17 mm Hg, *p* < 0.001), whereas the blood pressure of the rats in the SHD (SS + High salt diet + Daidzein) group was lower than that in the SH group (155.43 ± 2.93 *vs.* 174.71 ± 2.21 mm Hg, *p* < 0.001). 

During the intervention, systolic blood pressure steadily increased during the first three weeks. SS rats fed with a high-salt diet had significantly higher blood pressure than those in the other groups (Table 2), while daidzein treatment decreased the blood pressure.

### 2.2. Characteristic Response to Dietary and Drug Intervention

The 24 h urinary sodium excretion in the SH group was increased upon high sodium diet treatment in contrast to that of the SN group (28.73 ± 1.59 *vs.* 3.34 ± 0.62 mmol/day, *p* = 0.002), but decreased compared with the BH group (28.73 ± 1.59 *vs.* 41.15 ± 0.80 mmol/day, *p* = 0.023); while sodium excretion in the SHD group increased in contrast to that of the SH group (39.00 ± 0.82 *vs.* 28.73 ± 1.59 mmol/day, *p* = 0.047). Sodium excretion was also increased in SS.13BN rats after salt loading (3.86 ± 0.19 *vs.* 41.15 ± 0.80 mmol/day, *p* < 0.001) (Table 3). The 24 h urinary protein, 24 h urine volume and microalbuminuria in SS and SS.13BN rats fed with high sodium diet were higher than those fed with normal diet, but 24 h urine volume was increased in the SHD group compared with that in the SH group. In DS rats, creatinine clearance was decreased compared with that in the SN, BH and SHD group. Serum sodium was increased in both DS and SS.13BN rats. The SH group had higher kidney weight than the SN group and retained the same differences when corrected for body weight.

### 2.3. Impact of Different Interventions on the Expression of ERRα in the Kidneys of SS.13BN and Dahl Salt-Sensitive (DS) Rats

**Figure 1:**
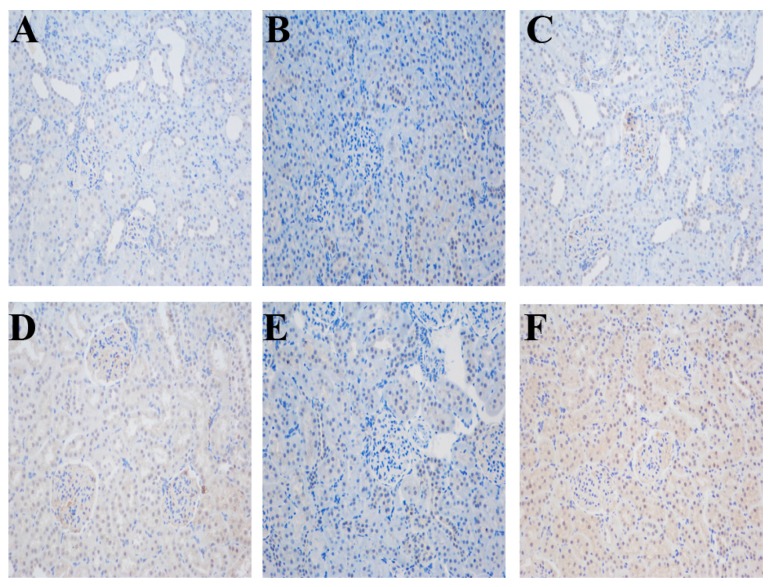
Immunohistochemical staining of estrogen-related receptor α (ERRα) in rat kidney tissues (original magnification ×200). (**A**) BN (SS.13BN + Normal diet) group; (**B**) BH (SS.13BN + High salt diet) group; (**C**) BHD (SS.13BN + High salt diet + Daidzein) group; (**D**) SN (SS + Normal diet) group; (**E**) SH (SS + High salt diet) group; and (**F**) SHD (SS + High salt diet + Daidzein) group. Nuclei were stained blue with Hematoxylin, and diaminobenzidine (DAB) was brown.

**Figure 2:**
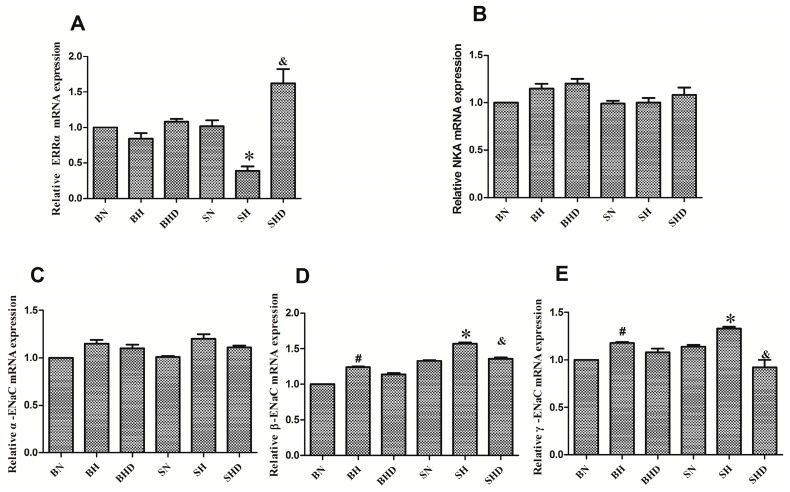
Expressions of ERRα, Na^+^/K^+^-ATPase, α-epithelial sodium channel (α-ENaC), β-ENaC and γ-ENaC mRNA after intervention. * *p* < 0.05, compared with BH, SN, and SHD groups. ^#^ *p* < 0.05, compared with BN group. ^&^ *p* < 0.05, compared with BHD group. (**A**) relative ERRα mRNA; (**B**) relative Na-K-ATPase mRNA; (**C**) relative α-ENaC mRNA; (**D**) relative β-ENaC mRNA; (**E**) relative γ-ENaC mRNA.

**Figure 3:**
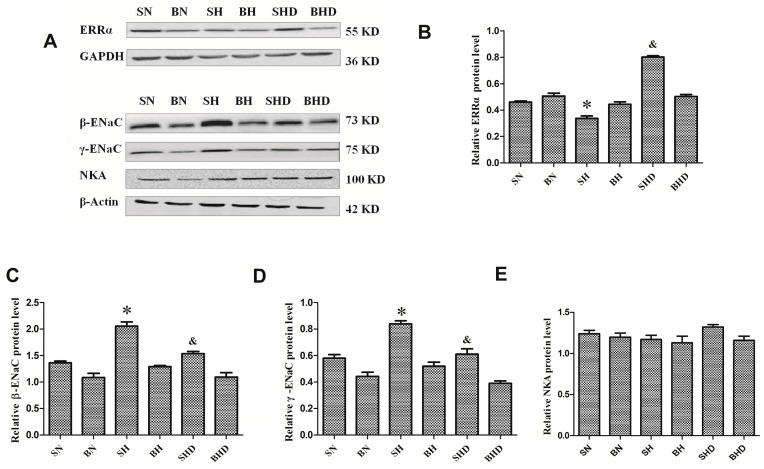
Expressions of ERRα, Na-K-ATPase, β-ENaC and γ-ENaC in rats. * *p* < 0.05, compared with BH, SN, and SHD groups. ^&^ *p* < 0.05, compared with BHD group. (**A**) protein expressions in all groups; (**B**) relative quantitative analysis of ERRα protein level; (**C**) relative quantitative analysis of Na-K-ATPase protein level; (**D**) relative quantitative analysis of β-ENaC protein level; (**E**) relative quantitative analysis of γ-ENaC protein level.

Chromatin immunoprecipitation (IHC) showed that the ERRα proteins were located in the kidney cortex of the rat, especially in renal tubules (Figure 1). After salt intervention, the mRNA expression of ERRα decreased in DS rats compared with that in the normal salt diet group and the BH group, although no significant difference was found between the BN group and the BH group, ERRα expression in the SHD group was higher than that in the SH group (Figure 2A). The protein expression of the ERRα also decreased after salt intake in SH group compared with those in the BH and SN groups (Figure 3B), and the quantitative value of ERRα in the SHD group was the highest among all groups.

### 2.4. The mRNA and Protein Expressions of Na^+^/K^+^-ATPase and Epithelial Sodium Channel (ENaC)

The mRNA expression of α-ENaC in both DS rats and the SS.13BN rats increased after salt loading, yet no significant difference was observed between rats treated with normal and high salt diets (Figure 2C). The expressions of β-ENaC and γ-ENaC mRNA (Figure 2D,E) and protein (Figure 3C,D) were elevated by high sodium diet in both DS rats but were decreased in the SHD group compared with those in the SH group. Although β-ENaC and γ-ENaC mRNA expressions were increased among the SS.13BN rats after high salt intervention, no significant difference was observed by Western blot analysis. There was no significant difference in the expression of Na^+^/K^+^-ATPase sodium excretion in DS rats or SS.13BN rats (Figure 2B and Figure 3E).

### 2.5. Chromatin Immunoprecipitation Assay (CHIP)

**Figure 4:**
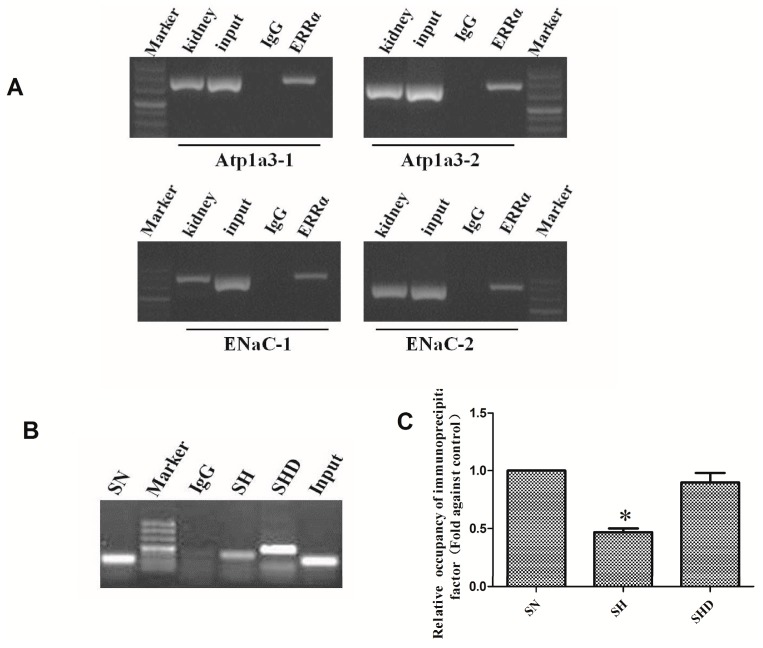
ERRα bound to Na^+^/K^+^-ATPase (*Atp1a*) and ENaC promoters in the kidney of the SS rats. (**A**) CHIP assay showing ERRα binding to the Atp1a and the ENaC promoters; (**B**) CHIP assay showing the relative binding capacity of the immunoprecipitated ERRα to the ENaC promoter in DS rats; (**C**) relative binding capacity of immunoprecipitated factor. Data were estimated by $\hat{2}$ (*C*_t_^mock^ − *C*_t_^specific^), and normalized with input, * *p* < 0.001, compared with SN and SHD groups. The SN group was used as the control.

The CHIP assay showed neither nonspecific precipitation nor PCR contamination from the negative controls (Figure 4). Positive PCR signals were observed in the samples treated with ERRα antibody, which indicated that ERRα was bound to the Atp1α and the ENaC promoters. Combined with the results of the qRT-PCR assay, the results of the CHIP assay indicated that high salt diet decreased gene expression by the binding of ERRα to the *ENaC* promoter, while daidzein treatments increased the relative binding capacity of ERRα.

## 3. Discussion

Kidneys play an important role in the development of salt-sensitive hypertension. Previous studies have shown that in salt-sensitive patients and Dahl salt-sensitive rats, renal reabsorption of sodium and water leads to a salt-sensitive form of hypertension [17]. In this study, for the first time, we showed that ERRα played an important role in the regulation of blood pressure in DS rats. In particular, ERRα, as a transcription factor, was bounded with the ENaC promoters and adjusted the reabsorption of sodium to regulate blood pressure.

Dahl rat is a low renin, salt-sensitive and hypertensive animal model. After being fed with a high salt diet (8% NaCl) for 3–4 weeks, the rats had dramatic increase in blood pressure [18]. Consistent with this result, our work showed that DS rats fed with high salt diet had a higher SBP because Dahl salt-sensitive rats were defective in renal sodium metabolism, which led to hypertension and obesity [19].

Clinical and animal studies have confirmed that most salt sensitive subjects have defects in renal natriuresis [20,21,22]. Factors associated with sodium retention include (1) decrease in ultrafiltration coefficient or single-nephron glomerular filtration rate, resulting in reduced sodium filtration; (2) increased sodium reabsorption resulted from increased expressions of vasoconstrictors such as angiotensin II, aldosterone and hyperactive sympathetic nervous system, or from decreased expression of vasodilators such as nitric oxide, kallikrein, dopamine and vasodilatory prostaglandins; as well as (3) altered regulation or expression of sodium channels in the renal tubules [23]. The results of this study showed that the sodium reabsorption of Dahl salt sensitive rats increased, and that there was obvious sodium retention in salt sensitive rats; while agonists of ERRα could increase the urinary sodium excretion. This implied that ERRα had a diuretic effect. We also found that the expressions of β-ENaC and γ-ENaC mRNAs in SS and SS.13BN rats increased after a high-salt diet intervention but decreased in the SHD group compared with those in the SH group. While the expressions of β-ENaC and γ-ENaC proteins increased only in the DS rats after salt intervention but decreased in the SHD group. The results of this study showed that the mRNA and protein levels of β-ENaC and γ-ENaC in the SS.13BN rats were not concordant, likely because they were regulated at the post transcriptional level [24]. This result is consistent with the previous observation that ENaC increases the sodium reabsorption [4,25,26,27,28]. In our study, the expression of Na^+^/K^+^-ATPase was not significantly different between DS rats and SS.13BN rats treated with either a low-salt or a high-salt diet. This was probably due to marinobufagenin (MBG), an endogenous ligand of α1 Na^+^/K^+^-ATPase (NKA), and reduced renal NKA activity. Plenty of evidence has shown that MBG is associated with the preferential inhibition of the sodium pump in the kidneys, resulting in sodium retention and pressor response in DS rats [29,30,31,32]. This is the limitation of our work and future studies are required.

Estrogen related receptor (ERR) is an important orphan member of the nuclear receptor superfamily, which is expressed in a wide range of tissues including heart, kidney and brain, especially in renal tubules [33,34]. Although there is no natural ligand, ERRs can be activated by non-hormonal signal [35]. Nevertheless, ERRs do not bind to estrogen and other known endogenous agonists [8]. The ERR family consists of three members, ERRα, ERRβ and ERRγ, and ERRα is expressed at high levels in kidneys. Hu *et al.* [36] have found that the ERRα protein level was significantly decreased in failing human hearts. According to a recent telemetry analysis study [15,37], ERRα expression is required for sustaining blood pressure of mice during the period of nocturnal activity. Daidzein, an ERRa agonist, binds to the ligand-binding pocket of ERRa, and constitutively activates gene transcription. DS rats subject to intragastric administration of daidzein had significantly lower SBP than the SH group. Giguere *et al.* [15] have found that ERR null mice are deficient in Na^+^/K^+^ handling with a mechanism favoring Na^+^ retention, and that the ERRα-dependent transcriptional program in the kidneys comprise several genes, including those involved in K^+^ and Na^+^ homeostasis (*Bsnd*, *Kcnq*, *Atp1*, *Scnn1a*, *Sgk2*, *Ghr*, *Gcgr*, *Lepr*, *Agt*, *Ace2*, and *Ren1 etc.*). Our study showed that the mRNA and protein expression of ERRα decreased in both SS.13BN and SS rats after a high-salt diet. This result indicated that ERRα could affect sodium excretion. More importantly, *in vivo* in chip assay indicated that ERRα bound with *ENaC* promoters. The results of CHIP and qRT-PCR assays indicated that high salt diet decreased the binding capacity of the *ENaC* promoter to ERRα, while daidzein treatments increased the relative binding capacity of ERRα. In the SHD group, the expression of ERRα was increased, the expressions of β-ENaC and γ-ENaC were decreased compared with those in the SH group, and the 24 h urinary sodium excretion was higher than that of the SH group. All these results indicated that ERRα reduced Na^+^ reabsorption and led to renal Na^+^/K^+^ handling disorders, and that it was correlated with the reduced expression of ERRα to regulate the expressions of β-ENaC and γ-ENaC and the balance of sodium and water. To summarize, high salt diet inhibits the expressions of β-ENaC and γ-ENaC by reducing the expression of ERRα, improves Na^+^ reabsorption and raises blood pressure. In contrast, daidzein promotes the expression of ERRα, thereby inhibiting the expressions of β-ENaC and γ-ENaC and lowering the blood pressure.

In summary, this report revealed the underlying mechanisms of salt sensitive hypertension. A high salt diet leads to increased renal 24 h urinary sodium excretion, which is associated with the decreased expression of ERRα. This affects the expressions of the target genes *β-ENaC* and *γ-ENaC* involved in the regulation of the reabsorption of water and sodium.

## 4. Materials and Methods

### 4.1. Experimental Animals and Protocols

Eight-week-old male Dahl salt-sensitive rats and SS.13BN rats weighed 262 to 298 g and were purchased from the Charles River Laboratories International, Inc. (Wilmington, MA, USA). SS.13BN rat is a salt insensitive control rat model derived from Dahl, with its chromosome 13 replaced by chromosome 13 of the Brown Norway rat. SS.13BN has 98% homology with the DS rats. Each group consisted of eight rats. The DS rats were divided into three groups: SN group (normal diet, 0.3% NaCl), SH group (high salt diet, 8% NaCl) and SHD group (high salt diet, 8% NaCl + Daidzein [38] (agonists of ERRα; 50 mg/kg/day). Similarly, the SS.13BN rats were also divided into three groups: BN group (normal diet, 0.3% NaCl) and BH group (high salt diet, 8% NaCl) and BHD group (high salt diet, 8% NaCl + Daidzein). Rats were housed in metabolic cages. The 24 h urine output and sodium/ potassium excretion data were collected. At the end of week 6, the rats were sacrificed, and plasma from the abdominal aorta was collected. Then, the kidneys were removed, flushed with saline, quickly weighed, snap-frozen in liquid nitrogen, and kept at −80 °C. All protocols were approved by the Institutional Animal Ethics Committee of Xi’an Jiaotong University (XJTU1AF2014LSL-023).

### 4.2. Measurement of Blood Pressure and Body Weight

The systolic blood pressure of the rats was measured once a week using the tail-cuff plethysmography with a computerized system (The BP-2000 Blood Pressure Analysis System™, Visitech Systems, Inc., Apex, NC, USA). A week before blood pressure measurement, the rats were subject to adaptive training in order to reduce measurement errors. Systolic blood pressure was measured five times at intervals of 2–3 min, and the average value was calculated. Body weights were also measured once a week for three times and averaged.

### 4.3. Measurement of Blood and Urine Biochemical Indicators

Before the end of the intervention, each rat was placed individually in a metabolic cage and provided with drinking water freely to collected 24 h urine. Then, the 24 h urine samples were sent to the hospital laboratory for testing. The rats were anesthetized with 10% chloral hydrate at the end of the intervention and supine fixed on the operating table. The abdominal aorta were exposed and separated under direct vision conditions, and 3–4 mL blood was collected through a vacuum vein blood collection needle inserted into the abdominal aortic root. The blood samples were centrifuged, and the supernatants were sent to the hospital for laboratory testing. The serum and urine biochemical indicators were examined by a Hitachi7060 automatic biochemical analyzer (Hitachi, Tokyo, Japan), and then creatinine clearance values were calculated as below: creatinine clearance = urine creatinine concentration (μM)/serum creatinine concentration (μM) × urine volume per minute (mL/min).

### 4.4. Immunohistochemistry

After the intervention, the rats were anesthetized with intraperitoneal injection of 10% chloral hydrate (3 mL/kg). The kidneys were flushed with saline after the abdominal cavities were open, fixed with neutral formalin for 30–50 min, embedded in paraffin, and sectioned at 5-µm thickness. Paraffin sections were dewaxed and hydrated, and then antigen was mixed with EDTA antigen retrieval buffer (pH 9.0). Three percent hydrogen peroxide was used to block the activity of endogenous peroxidase. The sections were dehydrated, blocked in 3% bovine serum albumin (BSA) for 30 min, and then incubated with anti-ERRα (1:1000; Abcam, Cambridge, UK). Sections were flatted on a humid chamber at 4 °C overnight, and then washed three times in phosphate buffered saline (PBS, pH 7.4) for 5 min each. Secondary antibody horseradish peroxidase (HRP labeled) were added and incubated with the sections for 50 min. Freshly prepared solution of diaminobenzidine (DAB) (K5007, DAKO, Glostrup, Denmark) was added and then rinsed with tap water to terminate coloration. Sections were stained with hematoxylin for 3 min, rinsed with tap water and examined under a microscope after dehydration.

### 4.5. QRT-PCR for ERRα, Na^+^/K^+^-ATPase and α-, β-, γ-ENaC Expressions in Kidneys

A total of 50 mg renal cortex tissues were homogenized. RNA was extracted using Trizol (Invitrogen, Carlsbad, CA, USA) and the purity and concentration of RNA were determined by UV spectrophotometry. Reverse transcription of RNA was performed using the RevertAid™ First Strand cDNA Synthesis Kit (Fermentas, Burlington, ON, Canada) according to the instructions. A 20 μL reaction was used for qPCR in an iQ5 real-time PCR detection system (Bio-Rad Laboratories, Hercules, CA, USA). The cycling parameters were 3 min at 95 °C, followed by 40 cycles of 10 s at 95 °C, 30 s at 55 °C and 30 s at 72 °C. GAPDH was used as the internal control. The results were analyzed using the 2^−ΔΔ*C*t^ method, ΔΔ*C*_t_ = [*C*_t_ (sample) − *C*_t_ (GAPDH)] − [*C*_t_ (BN group) − *C*_t_ (GAPDH)]. The primer (Takara, Shiga, Japan) sequences are shown in Table 4.

### 4.6. Western Blot Analyses of ERRα, Na^+^/K^+^-ATPase, and β-, γ-ENaC Expressions in Kidneys

A total of 50 mg renal cortex tissues previously stored at −80 °C were homogenized in a pre-cooled homogenizer in 800 µL radio immunoprecipitation assay (RIPA) with 2 mg/mL phenylmethanesulfonyl fluoride (Genshare, Shannxi, China), and then left to sit for 30 min on ice. The supernatant was collected after centrifugation at 14,000 rpm for 15 min at 4 °C. The protein concentration was determined using the BCA Protein Assay Kit (Beyotime Institute of Biotechnology, Haimen, China). The samples (45 µg) were separated on 12% polyacrylamide gel electrophoresis (SDS-PAGE) gels and transferred to polyvinylidene difluoride (PVDF) membranes at 250 mA for 2 h. Membranes were blocked in 5% skim milk powder dissolved in tris buffered saline Tween (10 mM Tris-HCl pH 8.0, 150 mM NaCl, and 0.05% Tween-20) at room temperature for 2 h. Then, the membranes were incubated in antibody of ERRα (1:1000; Abcam, Cambridge, UK), Na-K-ATPase (1:1000; CST, Beverly, MA, USA), β-ENaC (1:1000; Proteintech, Chicago, IL, USA), or γ-ENaC (1:1000; Abcam), followed by the addition of the horseradish peroxidase-conjugated anti-rabbit antibody (1:5000, Thermo Scientific, Waltham, MA, USA). GAPDH (1:5000, Bioworld, Louis Park, TX, USA) and β-actin (1:3000, Bioworld) antibodies were used to detect the corresponding expressions of these two genes as loading controls. Signals were quantified with the Bio Rad-IQ5 Image software (Bio-Rad Laboratories, Hercules, CA, USA).

### 4.7. Chromatin Immunoprecipitation (CHIP) Assay

The renal cortex of the kidneys of the rats was isolated and nuclear extracts were seeded onto 2 cm × 10 cm glass plates. Then, 10 mL media were added to the glass plate. When cell density reached 70%–80%, the rat kidneys were cross-linked with formaldehyde. The digested cells were counted and the number of cells in each dish was 1 × 10^7^/plate. The media were then replaced, and 270 µL of 37% formaldehyde were added into each Petri dish to a final concentration of 1% to crosslink the chromatin at 37 °C for 10 min. Cells were scraped and collected in 15 mL centrifuge tubes and then centrifuged at 2000 rpm for 3 min. The cell lysate was divided into 3 tubes. Cells were then lysed in 900 µL SDS lysis buffer containing protease inhibitors (PMSF, aprotinin), and genomic DNA was fragmented on ice by ultrasonication (output: 5 W; gap 15 s, work 10 s) into 500–1000 bp. Then, 80 µL of the lysates were removed and kept frozen at −70 °C. The DNA–ERRα complex was immunoprecipitated with rat anti-ERRα IgG (1:1000; Abcam, Cambridge, UK) in ice bath, followed by the addition of protein A agarose/salmon sperm DNA. The agarose/antibody/DNA proteins were collected. Then, 0.5 M EDTA (10 µL), 20 µL Tris-HCl (1M) and 2 µL proteinase K (10 mg/mL) were added to the supernatant to isolate the DNA and remove the protein. One part of the purified DNA was used as template for real-time PCR (RT-PCR) with specific primers (Table 4, c-Atp1a3 and c-ENaC). After PCR, the amplification product was recovered, cloned into the PMD-18T vector, and sequenced [39]. Another part of the DNA was used as the template for quantitative real-time PCR (qRT-PCR) with c-ENaC-2 specific primers targeting the ENaC gene in the DS rats. The relative binding capacity of a immunoprecipitation factor at a locus was estimated as $\hat{2}$ (*C*_t_^mock^ − *C*_t_^specific^), and normalized with input DNA, which was defined as 1.00, where *C*_t_^mock^ is the mean threshold cycles in qRT-PCR for mock samples, which was immunoprecipitation of ERRα by rabbit anti-IgG [37].

### 4.8. Statistical Analysis

Statistical analysis was performed with the SPSS 13.0 software (SPSS Inc., Chicago, IL, USA). All data are presented as mean ± SEM. The differences in blood pressure between the two groups were calculated by analysis of variance with the repeated measures design. Other data were analyzed by one-way analysis of variance (ANOVA) or *t*-test as appropriate. *p* < 0.05 was considered statistically significant. 

## Figures and Tables

**Table 1:** Effect of intervention on weight and systolic blood pressure (SBP, *n* = 8).

Group	Weight (g)		SBP (mm Hg)	
	Baseline	Intervention	Baseline	Intervention
BN	266.00 ± 5.18	340.60 ± 2.82	123.57 ± 1.67	144.71 ± 2.15
BH	262.63 ± 4.90	309.13 ± 6.05 ^#^	122.14 ± 2.61	146.43 ± 3.17
BHD	262.83 ± 1.92	301.00 ± 1.77	120.67 ± 1.20	147.67 ± 1.80
SN	268.17 ± 3.87	344.83 ± 8.64	121.29 ± 2.60	146.14 ± 2.88
SH	273.86 ± 5.57	313.4 ± 5.63 **	125.00 ± 3.00	174.71 ± 2.12 *
SHD	263.00 ± 2.86	306.56 ± 4.04	125.43 ± 2.03	155.43 ± 2.93

Data are presented as mean ± standard error (SEM), BN: SS.13BN + Normal diet; BH: SS.13BN + High salt diet; BHD: SS.13BN + High salt diet + Daidzein; SN: DS + Normal diet; SH: SS + High salt diet; SHD: SS + High salt diet + Daidzein. * *p <* 0.05 *vs.* SN, SHD and BH groups; ** *p <* 0.05 *vs.* SN group; ^#^ *p* < 0.05 *vs.* BN group.

**Table 2:** The SBP in male rats after 6 weeks of diet.

Group	Baseline	1 Week	2 Weeks	3 Weeks	4 Weeks	5 Weeks	6 Weeks
BN	123.57 ± 1.67	133.00 ± 2.42	135.86 ± 2.21	137.14 ± 2.65	131.29 ± 1.76	136.71 ± 3.03	144.71 ± 2.15
BH	122.14 ± 2.61	136.29 ± 1.84	140.71 ± 2.42	142.14 ± 2.20	140.71 ± 1.85 ^#^	144.29 ± 2.34 ^#^	146.43 ± 3.17
BHD	120.67 ± 1.20	125.67 ± 1.63	131.50 ± 0.85	134.67 ± 1.56	143.00 ± 2.08	142.67 ± 2.16	147.67 ± 1.80
SN	121.29 ± 3.00	132.86 ± 2.76	146.57 ± 2.09	144.71 ± 3.23	145.86 ± 2.30	145.29 ± 3.02	146.14 ± 2.88
SH	125.00 ± 3.00	142.86 ± 2.11 *	150.71 ± 2.67 ^$^	167.86 ± 3.07 *	161.29 ± 1.91 *	166.43 ± 3.29 *	174.71 ± 2.12 *
SHD	125.43 ± 2.03	132.29 ± 2.30	145.57 ± 2.63 ^&^	152.00 ± 2.73 ^&^	152.00 ± 2.82 ^&^	158.86 ± 1.10 ^&^	155.43 ± 2.93 ^&^

Data are presented as mean ± SEM, * *p <* 0.05 *vs.* SN, SHD and BH groups; ^$^ *p <* 0.05 *vs.* BH group; ^#^ *p* < 0.05 *vs.* BN group; ^&^ *p <* 0.05 *vs.* BHD group.

**Table 3:** Indicators in the serum and urine of SS.13BN and SS rats after intervention.

Parameters	BN	BH	BHD	SN	SH	SHD
Left kidney weight (g)	1.16 ± 0.03	1.21 ± 0.04	1.17 ± 0.04	1.15 ± 0.02	1.28 ± 0.03 **	1.2 ± 0.08
KW/BW (‰)	3.40 ± 0.09	3.80 ± 0.10 ^##^	3.84 ± 0.13	3.44 ± 0.07	4.36 ± 0.11 **^,$^	4.05 ± 0.23
Blood parameters (μmol/L)						
Serum creatinine	50.13 ± 3.16	47.93 ± 2.36	46.28 ± 0.46	53.35 ± 3.47	43.55 ± 2.12 **	48.33 ± 1.27
Serum sodium	137.35 ± 0.43	139.40 ± 0.90 ^#^	136.13 ± 0.14	138.80 ± 0.36	140.53 ± 0.68 **	138.88 ± 0.60
Serum potassium	4.50 ± 0.04	4.62 ± 0.04	4.65 ± 0.13	4.44 ± 0.05	4.37 ± 0.04 ^$^	4.86 ± 0.04
Urinary parameters						
24 h urinary sodium (mmol/day)	3.86 ± 0.19	41.15 ± 0.80 ^##^	55.46 ± 2.32	3.34 ± 0.62	28.73 ± 1.59 *	39.00 ± 0.82 ^&^
24 h urinary potassium (mmol/day)	1.81 ± 0.07	5.57 ± 0.30 ^##^	3.21 ± 0.43	0.97 ± 0.05	9.44 ± 0.53 *	6.12 ± 0.40 ^&^
24 h urinary albumin (mg/day)	24.19 ± 3.10	195.63 ± 34.00 ^##^	182.24 ± 23.10	17.25 ± 6.43	234.85 ± 27.00 **	186.52 ± 30.41
24 h urine volume (mL)	9.00 ± 0.71	62.25 ± 5.17 ^##^	73.50 ± 3.62	8.00 ± 0.41	52.25 ± 5.84 ^&&^	65.75 ± 5.90
Microalbuminuria (mg/day)	0.71 ± 0.04	4.97 ± 0.23 ^##^	4.58 ± 0.73	0.46 ± 0.15	6.05 ± 0.61 **	5.65 ± 0.82
Creatinine clearance (mL/min)	0.83 ± 0.12	0.72 ± 0.11	0.82 ± 0.07	0.80 ± 0.07	0.43 ± 0.07 *	0.70 ± 0.05

Data are presented as mean ± SEM, * *p <* 0.05 *vs.* SN, SHD and BH groups; ** *p <* 0.05 *vs.* SN group; ^&&^ *p <* 0.05 *vs.* SN and SHD group; ^$^ *p <* 0.05 *vs.* BH group; ^#^ *p <* 0.05 *vs.* BN and BHD groups; ^##^ *p* < 0.05 *vs.* BN group; ^&^ *p <* 0.05 *vs.* BHD group.

**Table 4:** Primer sequences.

Gene	Primers	Sequence (5′-3′)	Primer Length (bp)
*GAPDH*	Forward primer	GGCACAGTCAAGGCTGAGAATG	22
	Reverse primer	ATGGTGGTGAAGACGCCAGTA	21
*ERRα*	Forward primer	GATGTGGCCTCTGGCTACCACTA	23
	Reverse primer	CGGACAGCTGTACTCGATGCTC	22
*Na-K-ATPase*	Forward primer	ACTGGCAGAGAACGGCTTCCT	21
	Reverse primer	TCCGCTGCTCATAGGTCCACTC	22
*α-ENaC*	Forward primer	TGGTACCGCTTCCATTACAT	20
	Reverse primer	AGCGACAGGTGAAGATGAAG	20
*β-ENaC*	Forward primer	CACACCAACTGTGTCTTCCA	20
	Reverse primer	CATAGTTGGGAGGTGGAGTG	20
*γ-ENaC*	Forward primer	TCCTTGTATGGGGTCAAAGA	20
	Reverse primer	GAGCACAATTGAAATCCCAC	20
*c-Atp1a3-1*	Forward primer	TCACTGAACACGCCTGCTAC	20
	Reverse primer	TCCTGCCTTCTGACTGGGAT	20
*c-Atp1a3-2*	Forward primer	GTACCACGAGCCTTGTCTCAT	21
	Reverse primer	TTCAACCAGAAGCTCAAAAACCAG	24
*c-ENaC-1*	Forward primer	CCAGCCCCTACTTCACCTG	19
	Reverse primer	GAAAAGAGAGACAGACTGACAGGAC	25
*c-ENaC-2*	Forward primer	GGAGCCAGTCAAACAGTCCG	20
	Reverse primer	CATGGACCCCTTAGGCGAG	19
